# Femoral Stem Version in Primary Hip Arthroplasty: Challenges and Advances in 3D Planning

**DOI:** 10.2106/JBJS.OA.25.00300

**Published:** 2026-07-24

**Authors:** Angelika Ramesh, Johann Henckel, Alister Hart, Anna Di Laura

**Affiliations:** 1 Department of Mechanical Engineering, University College London, London, United Kingdom; 2 Royal National Orthopaedic Hospital, Stanmore, United Kingdom; 3 Institute of Orthopaedic & Musculoskeletal Science, University College London, London, United Kingdom; 4 Cleveland Clinic London, London, United Kingdom

## Abstract

» Native femoral version (NFV) demonstrates inconsistent correlation with prosthetic femoral version (PFV) and should not be relied upon in isolation for femoral version planning in cementless total hip arthroplasty (THA).

» Final stem version is strongly influenced by intramedullary canal morphology, fixation mechanics, and stem design philosophy. Straight, tapered wedge, and diaphyseal-engaging stems demonstrate greater variability in PFV, whereas anatomic and calcar-guided short stems may better preserve NFV.

» Current planning methods inadequately characterise three-dimensional canal anatomy and fail to simulate canal-stem interaction, limiting accurate prediction of PFV.

» Intraoperative estimation methods remain inconsistent and highly dependent on surgeon experience. Robotic-assisted THA enables real-time PFV measurements and supports femur-first strategies to optimise combined anteversion, although current systems still rely on computed tomography templating without true canal–stem interaction modelling.

» Reliable PFV prediction will likely require next-generation planning platforms integrating internal canal anatomy, statistical shape modelling and artificial intelligence-driven modelling into planning workflows.

## Introduction

Total hip arthroplasty (THA) demand is projected to rise by ∼40% by 2060^1^, increasing the need for accurate planning and precise implant positioning to achieve optimal surgical outcomes. Three-dimensional computed tomography (3D-CT) has become a valuable tool for preoperative planning and postoperative assessment^2-4^.

Traditionally, femoral stem planning has relied on 2D templating^5^, which lacks axial information and cannot account for femoral torsion^6,7^. Femoral stem orientation in cementless THA is constrained by intramedullary canal anatomy, stem size, and design^8-11^. As a result, predicting prosthetic femoral version (PFV) remains difficult and is poorly integrated into current planning systems, with surgeons often estimating PFV intraoperatively to optimize stability and combined anteversion (CV).

Although 3D-CT allows for accurate femoral anteversion measurements, during planning, native femoral version (NFV) is erroneously used as a surrogate for PFV^8-10^, despite poor correlation. In addition, current software tools are predominantly surface-based; they evaluate external bone contours but rarely incorporate internal canal morphology. Existing planning tools do not model stem-canal or implant-bone interaction, limiting their ability to predict the stem’s final orientation^9,10^.

The aim of this review was to critically evaluate current PFV planning approaches, identify limitations, and outline future directions for improving stem anteversion planning.

## Methods Proposed for Planning/Predicting PFV

**Fig. 1:**
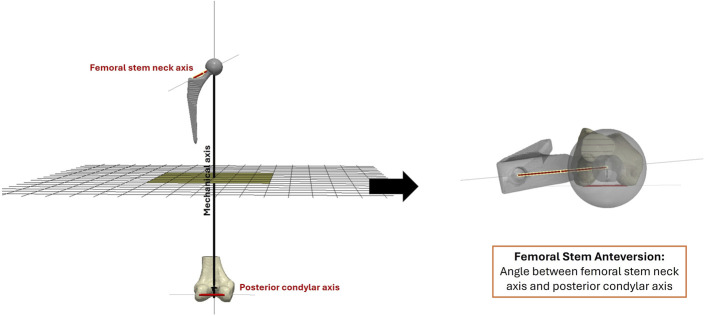
A systematic illustration depicting the definition and measurement of femoral stem anteversion within a standardized femoral coordinate system.

PFV (Fig. 1) planning has gained importance with the expanding use of press-fit stems^10^, and image-based computer-assisted navigation and robotic systems, yet reliable preoperative prediction remains an unmet need. Planning systems capable of identifying patients whose PFV may deviate significantly from intended ranges (∼15° ± 5°^11,12^) could help improve implant selection and reduce instability risk.

### 3D Digital Templating and Native Femoral Version

Some 3D-planning platforms and orthopaedic companies offer PFV planning, but based on the NFV, assuming this will be reproduced by the stem.

Hassani et al.^13^ assessed CT-based planning using HIP-PLAN (Symbios Orthopédie SA) but did not detail how anteversion was planned; NFV quantification preoperatively implied its central role in their planning approach. Imai et al.^14^ used the KYOCERA 3D Template software to assess NFV, planned version, and postoperative PFV, but provided limited description of how proximal femoral canal geometry influenced planning. Their earlier work^15^ showed good correlation (r = 0.80) between planned and postoperative PFV when using 3D computerized planning to estimate the version of metaphyseal fit-and-fill stems and then adjusting the acetabular cup to keep CV within the safe zone of 40° ± 15°^15^, though internal canal anatomy was not assessed. This highlights that the final component orientation in cementless THA also depends on stem design.

Inoue et al.^16^ retrospectively studied cementless THAs with short, fit-and-fill anatomical stems. Using the ZedHip (LEXI, Japan) software, the prosthetic components were navigated into position. However, “as a first step,” the femoral stem was adjusted to match the version and valgus angle of the native femur. Therefore, planned PFV was largely determined by NFV (r = 0.88). This approach is limited to stems allowing greater rotational freedom, such as short and/or anatomical stems designed for metaphyseal fixation. Sariali et al.^7^ also reported strong correlation between NFV and PFV (r = 0.84). However, modular neck adjustability warrants careful consideration of these results and complicates generalization.

**Fig. 2:**
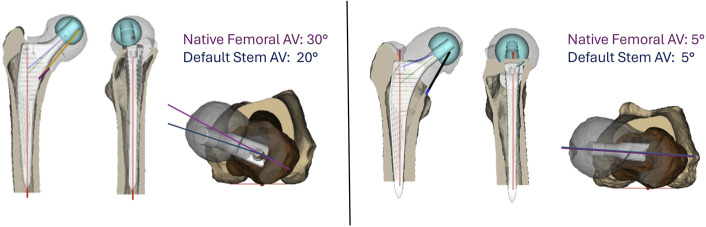
Two cases of 3D-CT–based femoral stem anteversion planning offered using the MyHip planning software (Medacta International SA), based on NFV measurements. NFV = native femoral version.

The Hip 3D software (mediCAD Hectec GmbH) similarly provides NFV measurements but does not guide surgeons toward target anteversion, as planning relies heavily on the preoperative femoral anteversion^17^. Several studies have shown NFV to be unreliable for predicting PFV, yet they are provided by several orthopaedic planning companies (Fig. 2—Medacta International SA^18^): Differences >15° have been observed by both Worlicek et al.^19^ and Moralidou et al.^9^. These findings emphasize the dominant influence of canal twist and flexion over external contours in determining PFV, suggesting planning methods should place less emphasis on the external anatomy. A summary of the studied literature comparing NFV with PFV is outlined in Table I.

**TABLE I:** Summary of Studies Evaluating Mismatch Between Native Femoral Version and Prosthetic Femoral Version in Uncemented Primary Total Hip Arthroplasty

Study	n	Femoral Prosthesis	Methodology	NFV-PFV mismatch (°)/mean ± SD (range)
Hassani et al.^13^	50	Symbios SPS (36 with modular neck)	CT-based 3D templating	0.6 ± 3.3 (−7 to 8)
Imai et al.^15^	65	PerFix 910 HA (Kyocera Medical)	CT-based 3D templating	7.7 ± 9.6 (−12.2 to 34.8)
Imai et al.^14^	118	PerFix 910 HA/J-Taper HA (Kyocera Medical)	CT-based 3D templating	NR
Inoue et al.^16^	65	APS Natural-Hip (Zimmer Biomet)	CT-based 3D templating	4.0 ± 3.6*
Sariali et al.^7^	223	SPS modular (Symbios)	CT-based 3D templating	Mean AV increase by 5.8°
Worlicek et al.^19^	55	Corail (Depuy)	Navigation-assisted THA	1.9 ± 9.5 (−20.7 to 21.5)
Moralidou et al.^9^	81	Quadra-H (Medacta)	CT-based 3D templating	Median 0.4 (−23 to 14)
Klim et al.^17^	130	ANA.NOVA (ImplanTec)/Corail (Depuy)	NFV and PFV assessed using 3D templating software	−0.8 ± 8
Marcovigi et al.^20^	362	MetaFix (Corin)	Robotic-assisted THA	1.6 ± 9.8 (−52 to 34)
Shao et al.^21^	117	Accolade II (Stryker)/S-ROM (Depuy)/Wagner Cone (Zimmer Biomet)	Robotic-assisted THA	−3.7 ± 14 (−41 to 35)
Sugano et al.^22^	60	Accolade II/Exeter (Stryker)	Robotic-assisted THA	4.2 ± 3.9 (−4 to 10)
Tsukeoka et al.^23^	50	M/L Taper Kinectiv (Zimmer Biomet)	Virtual THA simulation (implantation parallel to the T-line)	2.7 ± 6.0/3.5 ± 7.0 at osteotomy heights of 5 and 10 mm, respectively
Gold et al.^24^	736	N/A	Virtual landmark analysis on femoral neck cut plane	NR
Bargar et al.^25^	46	Fiber Metal Taper (Zimmer Biomet)	NFV and acetabular anteversion correlated with 3D templated femoral stem	8.7 ± 4.8 (1.8 to 20.5)^†^
Park et al.^26^	19	Linear stem (Enovis)	NFV measured at 5 levels	2.3 ± 5.9 (−10.2 to 11)^‡^
Sun et al.^12^	39	Accolade II (Stryker)	Robotic THA with NFV measured at 3 levels	0.8 ± 13.6 (−32.8 to 25.8)^‡^
Hu et al.^27^	133	Accolade/Secur-Fit (Stryker)	NFV at femoral head-neck junction measured and AV of anterior and posterior cortex measured at 7 levels	−7.5 ± 8.8
Marcovigi et al.^28^	102	Accolade II (Stryker)	Robotic-assisted THA and measured femoral metaphyseal AV at 3 levels	−9.8 ± 8.2
Weber et al.^8^	123	Corail (Depuy)	Radiographic iRatio analysis	NR

CT = computed tomography, ROM = range-of-motion, NFV = native femoral version, NR = not reported, and THA = total hip arthroplasty.

* Absolute values reported.

† Deviation from planned version.

‡ Lowest discrepancy method (midfemoral neck level).

Stem design and philosophy fundamentally influence mismatch between NFV and PFV due to differing fixation mechanics and dependence on intramedullary canal morphology and should therefore be considered when interpreting the aforementioned studies aiming to preserve NFV^17^.

Tapered wedge and straight stems, particularly those with narrow anteroposterior (AP) cross-sections and partial diaphyseal fixation, may permit greater rotational freedom and sagittal plane inclination, contributing to increased variability and reduced restoration of NFV. This may be a result of lower influence from proximal femoral anatomy^17,29-32^. By contrast, canal-filling and diaphyseal-engaging stems achieve greater cortical contact in the AP and mediolateral planes, constraining final stem orientation to the femoral canal. As the lower femoral neck and intertrochanteric region are typically more anteverted than the femoral head region, these stems may result in increased postoperative anteversion^33^. Longer diaphyseal-engaging stems may also “jam” earlier within the canal, further increasing mismatch.

Conventional straight stems have also been associated with reduced anteversion restoration and higher rates of postoperative retroversion compared with short-stem designs, which bears an increased risk of posterior dislocation^17,31,32,34^. To avoid varus malposition, surgeons often align these stems with the distal femoral axis, which may compromise anterior cortical fit, shift the head center posteriorly, and reduce PFV relative to NFV^17^. Straight stems are also more affected by factors such as anterior femoral bowing. By contrast, anatomical and calcar-guided short stems more reliably preserve anteversion through less distal/diaphyseal canal fill and improved proximal metaphyseal fit, fixing rotation in a position that cannot be easily modified^17,31^. The former also exhibits greater contact at the femoral neck cut level, possibly demonstrating greater influence of the femoral neck cut anteversion on the stem version and better match^35^. The latter achieves superior proximal fit guided by the preserved femoral neck’s calcar, helping restore NFV^17,31^. However, insufficient press-fit, poor bone stock, or undersizing can cause subsidence.

Other stem solutions include collared and cemented designs. Cementation enables manual control of anteversion and should be considered when intramedullary morphology risks excessive or insufficient version. Yet, identifying these outliers preoperatively poses a challenge due to limited characterization of the femoral canal.

### Intraoperative Estimation

Intraoperatively, PFV is traditionally estimated visually using landmarks on the proximal femur and knee-joint. Because uncemented stems allow restricted rotational adjustment, intraoperative estimation reflects achievable CV rather than true rotational control. A summary of studies comparing intraoperatively estimated stem version with achieved PFV is outlined in Table II.

**TABLE II:** Summary of Studies Evaluating Mismatch Between Intraoperatively Estimated and Achieved Prosthetic Femoral Version in Uncemented Primary Total Hip Arthroplasty

Study	n	Femoral Prosthesis	Methodology	Intraop Version-PFV Mismatch (°)/Mean ± SD (Range)
Lee et al.^36^	67	Taperloc (Zimmer Biomet)/M-stem (Corentec)/Tri-Lock (Depuy)	Goniometer estimation	2.0 ± 4.9 (−11.1 to 10.8)
Hirata et al.^37^	73	PerFix HA (Japan Medical Material)	Goniometer estimation	7.3 ± 5.7 (0-25)*
Dorr et al.^38^	99	APR/Alloclassic (Zimmer Biomet)	Visual estimation	NR
Wines and McNicol^39^	111	Synergy/Spectron (Smith and Nephew)	Visual estimation	−1.1 ± 10.4 (−25 to 30)
Pongkunakorn et al.^40^	114	Avenir/Excia (Zimmer Biomet)/Metha (Aesculap)	Visual estimation, protractor alone and protractor + spirit level	0.8 ± 3.7 (−7.1 to 8.0) using protractor and spirit level
Iwakiri et al.^41^	127	Taperloc (Zimmer Biomet)/Actis (DePuy)/Accolade II (Stryker)/Entrada (Ortho Development)	Novel PFV-measuring device	4.9 ± 3.9 (0-18.4)*
Unlu et al.^42^	59	F40 Press Fit (Biomet Europe)	Measured lesser trochanteric version and collo-femoral version preoperatively, and the operative collo-femoral version	NR
Sugano et al.^21^	60	Accolade II/Exeter (Stryker)	Robotic-assisted THA	2.1 ± 2.3 (−2 to 7) for cementless stems
				0.8 ± 1.8 (−2 to 5) for cemented stems

NR = not reported, PFV = prosthetic femoral version, and THA = total hip arthroplasty.

* Absolute values reported.

Validation studies of goniometer-based PFV measurements, performed with the lower leg vertical and knee axis horizontal, have demonstrated variable accuracy^36,37^, suggesting limited reproducibility of the technique in clinical practice. Importantly, measurement error seemed sensitive to lower-limb alignment and degenerative deformity, with both femorotibial angle^36^ and osteoarthritis (OA) severity^37^ identified as contributing factors. These findings indicate that the measurement reliability may deteriorate in patients with abnormal coronal alignment or advanced joint degeneration.

Conversely, Dorr et al.^38^ visually estimated femoral anteversion relative to the posterior thigh axis and found close agreement with postoperative CT, though results reflect the skill of an experienced surgeon. Poor precision has been found with visual estimation alone^39^ by other authors, with errors ranging from –25° to +30°, highlighting lower precision than instrument-guided methods. Pongkunakorn et al.^40^ evaluated a digital protractor with and without a spirit level; the combined approach produced the lowest error and was endorsed. Some have even constructed a dedicated anteversion-measuring device using a weighted chain and gravity-aligned marker^41^, although these devices have seen limited clinical validation and application, and reported discrepancies up to 18°. Thus, intraoperative PFV estimation cannot fully compensate for the canal-driven nature of stem positioning and may only guide safe CV.

### Robotic-Assisted THA

**Fig. 3:**
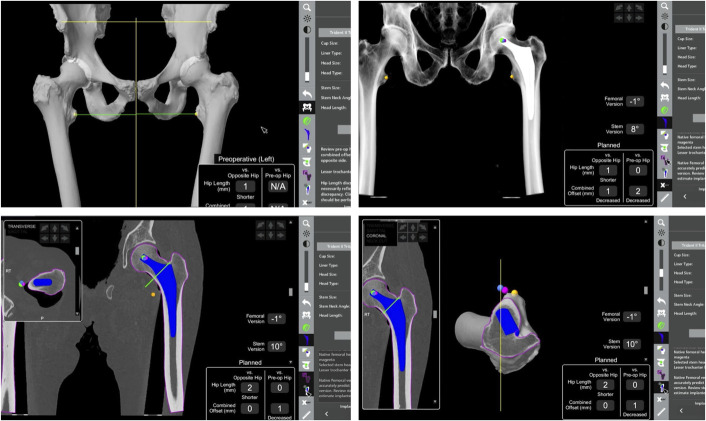
Stryker MAKO CT–based Total Hip Preplanning platform for a left THR. 3D digital templating is used to plan the stem size and orientation, to achieve the best fit within the femoral canal. Native femoral version is provided together with the stem version of the virtual implant (source: https://www.stryker.com/gb/en/joint-replacement/systems/mako-smart-robotics-overview.html).

The use of image-based robot-assisted technology in THA has helped enhance surgical precision and minimize visual estimation errors, with MAKO (Stryker) the most commonly used in hip and knee arthroplasty^43,44^. MAKO mandates preoperative CT scans for personalized planning and intraoperative registration of the patient’s anatomy^44,45^. Based on CT, virtual planning of cup position, leg length, femoral offset, and stem fit-and-fill within the medullary canal is possible. Using patient-specific 3D models, bony landmarks guide implant positioning and analysis of canal morphology informs stem selection (Fig. 3). The software also simulates joint range-of-motion (ROM) to prevent impingement.

A femur-first approach is preferable in robot-assisted THA, which requires reliable real-time measurement tools^19^. Intraoperatively, broach anteversion is measured in its final position by the robotic arm, to guide and dynamically adjust cup placement and achieve satisfactory CV (25°-50°)^14,36^.

Marcovigi et al.^20^ studied the difference between robotic-measured PFV and preoperative NFV, with a moderate correlation (r = 0.48). Other authors^21^ have found that intraoperative robotic-measured PFV measurements had the highest correlation (and greatest repeatability) with postoperative CT-based PFV compared with preoperative NFV, visually estimated, and goniometric stem version (r = 0.81). Some have even^22^ evaluated femoral stem anteversion accuracy using the MAKO THA system, comparing cemented Exeter and cementless Accolade II stems; the results confirmed that cemented stems achieved closer alignment to the CT-based plan than cementless stems. Robots therefore enable more accurate, real-time anteversion measurements but still underrepresent femoral canal morphology in preoperative planning, though stem fit-and-fill is partly considered.

### Computer-Simulation Studies

Two studies explored simulation of uncemented stem placement. Tsukeoka et al.^23^ virtually implanted straight stems parallel to the “T-line” (connecting the trochanteric fossa to the medial cortex midpoint) and found strong NFV-PFV correlation, suggesting the T-line as a guide for anteversion.

Gold et al.^24^ evaluated 13 anatomical axes at the neck resection level and measured the angle between each one and the transepicondylar axis. Findings showed that 8 axes had greater predictive accuracy for stem version compared with the lesser trochanter (LT) and proposed medial calcar points for intraoperative version prediction due to their accessibility.

These studies lacked postoperative validation, serving only as proofs of concept; their applicability remains primarily theoretical and merely focuses on surface landmarks rather than canal morphology.

### Alternative Approaches to PFV Prediction

Some studies propose alternative methods including external anatomical landmarks, radiographic ratios, and custom stems to address the challenge of predicting PFV. Unlu et al.^42^ assessed lesser trochanteric version but lacked postoperative PFV validation; this is a major limitation as the external anatomy poorly correlates with final stem anteversion, given the torsion of the canal along the femoral shaft^46^. On the other hand, Bargar et al.^25^ investigated whether acetabular anteversion could predict NFV and PFV; a poor correlation was found with both parameters.

Recognizing NFV’s unreliability, some studies^12,26-28^ explored the most accurate/optimal CT slice level for NFV measurement. For some, inconsistencies between PFV and NFV is believed to stem from inaccurate definitions of femoral neck version. Park et al.^26^ recommended the CT slice at the femoral head-neck junction; however, Sun et al.^12^ and Hu et al.^27^ found significant discrepancies between PFV and NFV measured at this level. The former instead advocated the midfemoral neck slice, whereas the latter proposed a sagittal 3-point fixation concept. Marcovigi et al.^28^ found metaphyseal axis anteversion (MAA) measured 10 mm above the LT best approximated PFV, outperforming MAA measured at higher levels and conventional NFV definitions.

2D radiographic methods for planning femoral stem anteversion remain uncommon due to magnification and projection errors^7,22^ and the influence of patient positioning during imaging. Nonetheless, Weber et al.^8^ proposed an isthmus Ratio (“iRatio”), correlating canal and calcar isthmus widths with postoperative PFV. This showed moderate correlation (r = 0.58), useful mainly for detecting extreme anteversion or retroversion rather than precise planning^8^.

With growing emphasis on patient-specific planning, custom stems (e.g., Individual Hip, Symbios Orthopédie)^47,48^ have been introduced to better match femoral canal morphology. These incorporate built-in version based on CT-derived morphology and show promise for preserving NFV while conforming to the unique intramedullary morphology.

### PFV and Its Deviation from Femoral Anteversion: Clinical Relevance

With femur-first strategies, compensating for abnormal PFV by adjusting cup anteversion may not always be feasible. Excessive cup adjustment can reduce host bone contact, create anterior overhang, and increase the risk of impingement or instability^34,49^.

A major aim of THA is to restore femoral anteversion or achieve PFV and CV within traditionally accepted safe zones. Deviation between PFV and NFV can affect postoperative outcomes: Femoral head displacement from native parameters can change muscle force vectors, narrow impingement-free ROM and increases the risk of dislocation, edge loading, and squeaking^17,50^. Excessive PFV raises the likelihood of anterior subluxation or posterior rim impingement during flexion-external rotation^51^. Reduced PFV also raises torsional moments on the stem, heightening loosening risk^52^. Anteversion changes can modify hip and foot rotation; increased NFV reduces external foot rotation and produces compensatory internal hip rotation during gait^11,53^.

Satılmış et al.^54^ found that DDH patients with preserved NFV had the best postoperative stability. Increased anteversion correlated with pain, instability, and knee dysfunction, while decreased anteversion offered moderate functional improvements but higher dislocation risk. Overall, PFV-NFV deviations affect stability, ROM, muscle mechanics, and gait, reinforcing the importance of accurate version planning.

### Future Directions and Opportunities

Although 3D-CT captures complete femoral anatomy, current planning focuses predominantly on external bone contours. As uncemented stems follow the intramedullary canal, overlooking the internal geometry limits PFV prediction. Existing 3D software rarely simulate interactions between the implant, canal, and bone, and most default to NFV-based planning. This review has underscored this gap.

Robotic-assisted systems demonstrate superior precision and repeatability in intraoperatively measuring PFV. However, surgeons have limited flexibility to alter implant design or fixation method at this stage. Preoperative planning still hinges on digital templating, limited in accuracy by its focus on NFV.

A major unmet need identified is preoperative identification of “at-risk” morphologies, such as highly twisted or bowed canals, where uncemented stems are likely to result in excessive or insufficient anteversion. Recent statistical shape modelling (SSM) studies have highlighted large variability in proximal and distal canal torsion^46^. Integrating SSM into planning platforms could help classify femoral canals into low-risk, medium-risk, and high-risk patterns for suboptimal PFV. Artificial intelligence (AI)–driven models trained on large imaging data sets could improve understanding of correlations and generate patient-specific and implant-specific PFV predictions.

Future platforms should combine surface anatomy, internal canal morphology, implant size and geometry, and fixation behavior to simulate stem positioning and predict version. Ultimately, planning tools will need to model true bone-implant interactions, offering recommendations on stem design, sizing, and fixation method when native morphology risks malversion.

## Conclusion

Unlike the acetabular cup, the femoral stem is constrained by the internal morphology of the proximal canal, which varies widely and does not correlate with external anatomy. This constraint produces suboptimal PFV in uncemented THA and makes accurate planning challenging.

This review highlights the erroneous and excessive reliance on NFV to predict PFV, and that current planning methods neglect internal canal geometry, limiting planning accuracy. Future planning should be patient-specific and technology-driven. Achieving accurate PFV planning will require advanced tools that incorporate internal anatomy into standard workflows, such as SSM, AI-driven modelling, and robotic-assisted systems. Progress in these areas is essential for accurate, reproducible PFV planning in THA and reducing risks of instability, impingement, and suboptimal biomechanics.

## Funding

The authors disclose no sources of funding supporting this project.
